# The Dream of God: How Do Religion and Science See Lucid Dreaming and Other Conscious States During Sleep?

**DOI:** 10.3389/fpsyg.2020.555731

**Published:** 2020-10-06

**Authors:** Sergio A. Mota-Rolim, Kelly Bulkeley, Stephany Campanelli, Bruno Lobão-Soares, Draulio B. de Araujo, Sidarta Ribeiro

**Affiliations:** ^1^Brain Institute – Federal University of Rio Grande do Norte, Natal, Brazil; ^2^Physiology and Behavior Department – Federal University of Rio Grande do Norte, Natal, Brazil; ^3^Onofre Lopes University Hospital – Federal University of Rio Grande do Norte, Natal, Brazil; ^4^The Sleep and Dream Database – Portland, OR, United States; ^5^Biophysics and Pharmacology Department, Federal University of Rio Grande do Norte, Natal, Brazil

**Keywords:** dreams, religion, meditation, lucid dream, out of body experiences

## Abstract

Lucid dreaming (LD) began to be scientifically studied in the last century, but various religions have highlighted the importance of LD in their doctrines for a much longer period. Hindus’ manuscripts dating back over 2,000 years ago, for example, divide consciousness in waking, dreaming (including LD), and deep sleep. In the Buddhist tradition, Tibetan monks have been practicing the “Dream Yoga,” a meditation technique that instructs dreamers to recognize the dream, overcome all fears when lucid, and control the oneiric content. In the Islamic sacred scriptures, LD is regarded as a mental state of great value, and a special way for the initiated to reach mystical experiences. The Christian theologian Augustine of Hippo (354–430 AD) mentions LD as a kind of preview of the afterlife, when the soul separates from the body. In the nineteenth century, some branches of the Spiritism religion argue that LD precedes out-of-body experiences during sleep. Here we reviewed how these religions interpret dreams, LD and other conscious states during sleep. We observed that while Abrahamic monotheisms (Judaism, Christianity, and Islam) recognize dreams as a way to communicate with God to understand the present and predict the future, the traditional Indian religions (Buddhism and Hinduism) are more engaged in cultivating self-awareness, thus developed specific techniques to induce LD and witnessing sleep. Teachings from religious traditions around the world offer important insights for scientific researchers today who want to understand the full range of LD phenomenology as it has emerged through history.

“Myths are public dreams, dreams are private myths”

Joseph Campbell—The power of myth (1988)

*“In the dream … we have the source of all metaphysic. Without the dream, men would never have been incited to an analysis of the world. Even the distinction between soul and body is wholly due to the primitive conception of the dream, as also the hypothesis of the embodied soul, whence the development of all superstition, and also, probably, the belief in god. ‘The dead still live: for they appear to the living in dreams.’ So reasoned mankind at one time, and through many thousands of years”* Friedrich Nietzsche—Human, all too human (1878)

## Introduction

The term “lucid dreaming” (LD) was coined by Van Eeden (1913), to describe a kind of dream during which “the reintegration of the psychic functions is so complete that the sleeper remembers day-life and his own condition, reaches a state of perfect awareness, and is able to direct his attention, and to attempt different acts of free volition” (Van Eeden, 1913, p. 446). According to LaBerge et al. (1986), LD would be simply “dreaming while being conscious that one is dreaming”. LD started to be objectively studied by the work of Hearne (1978) and LaBerge (1980a,b), who developed a technique that consists of instructing dreamers to move the eyes voluntarily to indicate that they became lucid. Despite these first scientific accounts being recent, LD have been described by various religions for a much longer time. In this opinion article, we review how Hinduism, Buddhism, Judaism, Christianity, Islam and Spiritism interpret dreams, LD and other conscious states during sleep.

## Hinduism

Hinduism has its origins in India approximately 3,500 years ago, and is called Sanâtana Dharma (in Sanskrit: 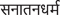), which means “The eternal law.” Like most ancient societies, Hindus saw dreaming as divine and prophetic, and one of the most reliable sources of insight (Freud, 1900; Ribeiro, 2019). Hindus interpret dreams and the whole world as illusions made by a God named Vishnu (Shulman and Stroumsa, 1999). In the mystical texts known as the Upanishads, dreaming becomes a personal experiential path toward the realization of the illusory nature of the self and all reality.

Interestingly, Hindus divide consciousness into waking, dreaming, and deep sleep, and believe that both dreaming and deep sleep are more important than waking. This is the opposite of Western culture, which considers waking as the main state–a synonym of “real,” sleep as just a complementary state, and dreaming as “unreal” (Bulkeley, 2008). Some Hindu practitioners believe that only in deep sleep can we be completely free from thoughts, but not during waking and dreaming. They also consider that there is a form of consciousness during deep sleep, but that it was not possible to have LD in this state (Sharma, 2006). In fact, studies have found that LD–objectively indicated by the eye movements technique (Hearne, 1978; LaBerge, 1980a,b; Mota-Rolim, 2020)–was already described during sleep onset (N1 stage), light sleep (N2), and rapid eye movement (REM) sleep, but not during deep sleep (N3) (LaBerge et al., 1981a,b; Stumbrys and Erlacher, 2012; Mota-Rolim et al., 2015; Baird et al., 2019). However, this is still debatable, especially when we consider the Hindu tradition of spiritual sleep: Yoga Nidra. Contemporary texts consider Yoga Nidra a kind of LD state, in which dream imagery takes place for the practitioner, who do not identify or become attached to them, remaining as an objective observer (Miller, 2005; Hoye and Reddy, 2016).

Also known as “Yogic Sleep,” the Yoga Nidra (Sanskrit: 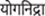) means “blissful relaxation” and is considered one path to achieve the state of Samâdhi or self-realization (Saraswati and Hiti, 1984). The Yoga Nidra is first mentioned in the Upanishads, which are part of the Vedas—the ancient Sanskrit texts that contain the oldest scriptures of Hinduism (Desai, 2017). Interestingly, the Yoga mantra OM/AUM refers to different consciousness states: “A” (awakening), “U” (dreaming), “M” (deep sleep). The fourth state, Turiya or the Transcendental state, is represented by the combination AUM (Sharma, 2018). In the Bhagavad Gita, one of the most sacred and revered Hindu texts, the God Krishna is in Yoga Nidra when the prince Arjuna first meets him: half-awake.

The founder of the modern practice of Yoga Nidra, divided it into eight steps that basically consist of paying attention to different parts of the body, to breath and to do visualizations while lying on the floor in shavasana (the corpse pose) to observe your mind reaction (Saraswati and Hiti, 1984). Actually, one of these steps called “rotation of consciousness” is a variation of the ancient Tantric practice of “Nyasa,” which means “to take the mind to that point” (Rani et al., 2011). However, there are also other ways of practicing Yoga Nidra, such as one described in the Himalayan tradition, which consists of using your breath to concentrate your attention on the Ajna (point between eyebrows), Vishuddha (throat), and Anahata (heart) chakras. It is said to be preceded by two preparatory practices called Shavyatra and Shitalikarana. In the first one, the attention travels through the body in 61 points. The term “shava” means “corpse” and “yatra,” “journey.” In the second, the breath travels from different parts of the body in a specific way. The term “shitalikarana” comes from the Sanskrit verb “shitalikaroti,” which means, “to cool or calm.” The Yoga Nidra is also considered the state of consciousness during the deep sleep, which is believed to lead to self-realization (Grouven, 2018).

One of the major debates in classical Indian philosophy is whether consciousness is present or not in deep sleep. The philosophical schools of Advaita Vedânta and Yoga affirm that consciousness is present in dreamless sleep, whereas the Nyâya School says it is not (Thompson, 2015). The term “witnessing sleep,” on the other hand, describes the co-existence of transcendental consciousness and sleep. According to Alexander (1988) and Travis (1994) there are three types of consciousness in sleep: LD; witnessing dreaming–an experience of quiet, peaceful inner awareness or wakefulness completely separate from the dream; or witnessing deep sleep–an experience of quiet, peaceful, inner state of awareness during dreamless sleep. Recent works also consider that there is a form of consciousness in dreamless sleep (Windt et al., 2016; Siclari et al., 2017). In one study with the yogi Swami Rama, scientists found that he would remember everything that had happened to him while in a state of Yogic Sleep–in which the EEG showed 40% of delta wave activity, which resembles deep sleep. He was able to recite 9 of the 10 sentences given to him while in that state (Ancoli et al., 2012), supporting the observation that information can affect us even when we are in an “unconscious” state (Ruch and Henke, 2020).

Another study obtained a similar result with a different technique called Transcendental Meditation, which uses mantras (Woolfolk, 1975) and shares the same goal with Yoga Nidra (Cranson et al., 1991). The authors found that 11 long-term practitioners were able to report being aware during sleep when compared to 9 short-term practitioners and 11 non-practitioners. EEG recordings showed that during deep sleep, the experimental group (long-term meditators) had greater theta-alpha activity simultaneously with delta activity and lower muscular tonus, when compared to the other groups (short-term and non-practitioners). The authors suggested that transcendental consciousness during sleep is distinct from LD, since the last one occurs almost exclusively during phasic REM and more often during later REM periods (Mason et al., 1997; Baird et al., 2019). Finally, studies on Mindfulness Meditation practices also provide empirical support for the possibility of a kind of consciousness in deep sleep (Tang et al., 2015). According to Thompson (2015), proficient meditators occasionally report “witnessing sleep,” when they experience no specific thought contents or imagery. These participants had differences in EEG activity during sleep when compared to non-meditators and inexperienced meditators, such as enhanced gamma-band activity (Mason et al., 1997; Ferrarelli et al., 2013; Dentico et al., 2016; Maruthai et al., 2016).

Another study found that the high-lucidity group disclosed increased gray matter volume in the frontopolar cortex (BA9/10) when compared with the low-lucidity group. Additionally, the blood oxygen level-dependent signal increased in this brain area during thought monitoring in both groups, and even more in the high-lucidity group. The authors suggest that metacognitive practices and LD share neural systems, in particular in the domain of thought monitoring (Filevich et al., 2015). It was also reported that the frequency of LD is more positively related to mindful presence state rather than to acceptance mind state. However, it remains unclear whether the relationship between mindfulness and LD is influenced by actual meditation practice other than individual predispositions (Stumbrys et al., 2015).

## Buddhism

Buddhism originated around 2,500 years ago in India, and today is divided in three branches: Theravada (The School of the Elders), Mahayana (The Great Vehicle), and Vajrayana (The Diamond Vehicle). The Vajrayana School was established in Tibet during the eighth century and gave origin to Tibetan Buddhism, which practices the Dream Yoga, in Sanskrit 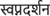
–a meditation technique focused on developing awareness during the dream state. Curiously, Buddha himself is known as “The Awakened” or “The Enlightened,” both related to the word “lucid,” as in LD (Rosch, 2014).

The dream yoga practice has four stages. However, before practicing the dream yoga, LaBerge (2003) describes two preparatory techniques. In the first one, the dreamer must recognize the dream as it unfolds, and some techniques such as meditating about it before going to sleep can help (LaBerge, 1980b). Then, the dreamer must try to overcome all possible fears when becoming lucid, aiming to prevent awakening–a common undesired outcome, especially in inexperienced lucid dreamers (Mota-Rolim et al., 2013). After these preparatory techniques, the dreamer can start the first stage, in which one must contemplate the dream, and reflect on how it is or not similar to real life, since both are illusions in constant changing, a fundamental concept of Buddhism. By this previous insight, the dreamer must then try to control the oneiric content. This stage is especially important to those who suffer from recurrent nightmares, because by becoming lucid during the nightmare the dreamer may learn not to be afraid, since nothing can cause real physical harm inside the dream. Other possibilities include transforming the nightmare in a good dream, or simply waking up (Mota-Rolim and Araujo, 2013; Macêdo et al., 2019). In the third stage, the dreamer must recognize that the dream body has not a material substance, and the same idea could be applied to other people or objects in the dream. Finally, in the fourth and last stage, the dreamer should try do visualize the deities, such as Buda, and then a revelation would happen (LaBerge, 2003).

There is a relation between the occurrence of LD and meditation practice (Gackenbach, 1981, 1990; Hunt, 1991; Mota-Rolim et al., 2013; Sparrow et al., 2018). One possible explanation is that experienced meditators have an increased density of rapid eye movements during REM sleep (Mason et al., 1997). This may enhance LD frequency because LD is related to phasic (activated) REM sleep, i.e., REM sleep periods with rapid eye movements (LaBerge, 1980a; LaBerge et al., 1981b, 1986). The neuropsychological mechanisms that underlie this finding is not yet clear, but may have to do with the fact that phasic REM sleep has an autonomic activation that resembles waking, and that LD seems to be a mixture (Voss et al., 2009) or a transition phase (Mota-Rolim, 2020) between REM sleep and waking. Another explanation is that LD increases alpha band (8–12 Hz) power (Ogilvie et al., 1982; Tyson et al., 1984; Mota-Rolim et al., 2008), as also observed in a relaxed wake state with eyes closed (Berger, 1929; Adrian and Matthews, 1934) and during meditation (Varela et al., 1945). Furthermore, meditative states of “focused attention” (Himalayan Yoga), “open monitoring” (Vipassana), and “open awareness” (Isha Shoonya Yoga), show increased global coherence in the gamma band (Vivot et al., 2020), as also observed during LD (Mota-Rolim et al., 2008; Voss et al., 2009). Greater capacity for mental control emerges in both experienced meditation practitioners and frequent lucid dreamers (Blagrove and Tucker, 1994; Blagrove and Hartnell, 2000). Finally, a connection between meditation and LD is through the development of metacognitive abilities, such as mindfulness (Filevich et al., 2015; Stumbrys et al., 2015). These various alternative explanations are not necessarily mutually exclusive.

The leader of Tibetan Buddhism today, Tenzin Gyatso, the fourteenth Dalai Lama, has supported western research on LD as a potential bridge between modern science and ancient religious wisdom. When asked to describe his views of LD, the Dalai Lama replied:

“There is said to be a relationship between dreaming, on the one hand, and the gross and subtle levels of the body, on the other. But it is also said that there is such a thing as a “special dream state.” In that state, the “special dream body” is created from the mind and from vital energy (prana) within the body. This special dream body is able to dissociate entirely from the gross physical body and travel elsewhere. One way of developing this special dream body is, first of all, to recognize the dream as a dream when it occurs. Then, you find that the dream is malleable, and you make efforts to gain control over it. Gradually, you become very skilled in this, increasing your ability to control the contents of the dream so that it accords to your own desires. Eventually it is possible to dissociate your dream body from your gross physical body”.

Another relevant Tibetan Buddhist yoga addresses useful techniques to achieve LD. In the so-called Tibetan sleep yoga, both witnessing sleep and LD are used to develop mind flexibility. Mind flexibility is, according to Wangyal and Dahlby (1998) a crucial characteristic to relativize the way things are in this world, and thus to better administer our feelings and attachment to things. As a consequence, cultivation of sleep witnessing in the beginning and LD at the end of the night may lead to favor paving the path to enlightenment. Thus, according to Wangyal, sleep yoga practitioners can collect useful fruits for enlightenment with this practice, being a reasonable alternative in relation to practices with no related emphasis and with no cultivation of special dreams. The advantage of these practices is emphasized in the way that the practitioner can train a transforming mind yoga technique even when you are in bed for sleep. In this way, Tibetan Yogis developed a specific classification of dreams, as: (1) ordinary dreaming (both lucid and non-lucid), (2) dreams of clarity (both lucid and non-lucid), and (3) clear light dreams (only appearing as LD). As reported by Wangyal and Dahlby (1998), dreams of clarity differences in relation to ordinary dreams rest on more stability of the practitioner, and on the rising of special images and traces that “present available knowledge directly from consciousness below the level of conventional self.” Finally, clear light dreams are a specific kind of dream which “occurs when one is far along the path.” This dream appears when the practitioner experiences non-dualistic mind states, and is also a non-dualistic dream: the practitioner “does not reconstitute as an observing subject in relation to the dream as an object, nor as a subject in the world of the dream,” integrated with the non-dual state.

In order to cultivate those lucid and non-lucid dream states, the practitioner is oriented to perform diverse yoga techniques, which include calm abiding meditation, mind flexibility routines during the day (such as imagination of the world and his/herself as a dream), awareness and remembrance of dreams and, during the sleep, visualization of Tibetan symbols (tingles), and syllables associated to parts of the body at four different moments during the night (Wangyal and Dahlby, 1998). Lucid dreams, in this tradition, are supposed to arise specially in the last part of the night, in a clear coincidence with the classical physiological occurrence of more robust REM episodes.

In this context, we argue that he scientific study of Tibetan sleep yoga practices could address relevant questions in neuroscience. With the use of EEG and functional anatomy scans, we may better understand the neural dynamics of these states in experienced practitioners, as well as in beginners. We may address each of the four visualization practices of this Tibetan sleep yoga during sleep, and get the comprehension of their neural signatures. Also, we may better understand in which way LD-related practices can influence neuroplasticity and if they can work as a mitigation technique for anxiety and depression states, or if it can be a useful tool in modern societies to develop a better emotional stability and self-control. As occurred with classical mindfulness techniques, which re-emerged from ancient Asian traditions to be adopted for lay practice in this turbulent twenty-first century world, a similar adaptation of Tibetan sleep and dream yogas (with possible neuroplasticity effects demonstrated by neuroscience studies), could add additional muscle to prevent the widespread occurrence of mental diseases in our present times.

These experimental approaches to dreaming, treating it as a realm of consciousness capable of being actively explored and intentionally cultivated, is very different from the approach developed in the traditional Abrahamic religions, as the next sections will show.

## Judaism and Christianity

Judaism originated approximately 3,800 years ago, when Abraham established a covenant with God. Christianity has its origins from the Judaism, about 2,000 years ago. Judaism and Christianity are monotheistic, and share common origins in the Old Testament. Judaism Signals of God can be obtained by visions, through voices, and, of course, through dreams. The approach of dreams in Judaism and Christianity is clearly distinct from those of the two traditional Indian religions mentioned above. In both Buddhism and Hinduism, dreams are used as tools for the expansion of consciousness and gain in self-control, as part of the path toward enlightenment, or mastering of body and mind (LaBerge, 1980b; Saraswati and Hiti, 1984). In Judaism and Christianity, dreaming serves primarily as a means of communication between humans and God. The dreams can take many forms—visual images, auditory commands, frightening nightmares—but the common feature is a revelatory message from the divine to the dreamer.

In the Old and the New Testaments, the word “dream” appears over a hundred times. Hebrews, Babylonians and ancient Egyptians shared traditions of dream interpretation. As exemplified in the interpretation of the Egyptian pharaoh’s dreams by Joseph (Gen 1–41), and in the interpretation of Nebuchadnezzar’ dream by Daniel (Daniel 2:43–45), the Jewish people were extremely successful in obtaining the grace of foreign rulers through the mastering of dream interpretation, with an impact on public policies. In Egypt, Joseph interpreted a dream report of seven fat cows eaten by seven gaunt cows as predictive of 7 years of abundance followed by 7 years of famine; and recommended the construction of silos to stock grains. In Babylon, Daniel interpreted the king’s dream about a huge statue hit by a stone, as a precognitive description of the future generations and kingdoms. However, despite the critical importance of dreams in biblical texts, we found no direct allusion to a LD.

The same is true of the references to dreams in the New Testament, which are fewer than in the Old Testament but convey the same basic theme of human-divine communication. In this theological context, LD appears less relevant because God’s messages can be effectively delivered in non-lucid dreams. Higher levels of consciousness within the dream state do not really matter; what matters is remembering the dream upon awakening and properly interpreting its divine significance. For example, in Numbers 12:6: “And he said, hear now my words: If there be a prophet among you, I the Lord will make myself known unto him in a vision, and will speak unto him in a dream.”

This difference is illustrated by the early Christian theologian Augustine of Hippo (354–430 AD) in a letter in which he mentions the LD experience of a friend who was doubting the doctrine of the eternal soul. In his dream an angelic young man appears and brings him to a state of lucid awareness:

“As while you are asleep and lying on your bed these eyes of your body are now unemployed and doing nothing, and yet you have eyes with which you behold me, and enjoy this vision, so, after your death, while your bodily eyes shall be wholly inactive, thee shall be in you a life by which you shall still live, and a faculty of perception by which you shall still perceive. Beware, therefore, after this of harboring doubts as to whether the life of man shall continue after death.” (quoted in Bulkeley, 2008, p. 181)

Augustine clearly recognizes the phenomenon of LD, of conscious self-awareness within sleep, and yet in his religious worldview it has a very different significance from the Hindu and Buddhist perspective. For Augustine, LD is a kind of preview of the afterlife, when the soul becomes completely separated from the body. The experience of LD confirms what Christians should already know. There is no interest here in exploring LD beyond those theological limits and probably this is why there is a lack of scientific works on these experiences in Christianity/Judaism.

## Islam

Members of the Islamic faith believe that the word of God (Allah) was revealed to humanity by the Prophet Muhammed in 610 AD, continuing and completing the revelations that begun in the Jewish and Christian religions. Importantly, this happened after the visit of the archangel Gabriel to Muhammed in what many believe was a dream (Hermansen, 2001). Moreover, before this first revelation, it is believed that Muhammed experienced many dreams full of spiritual meaning, which induced him to begin his preaching. In the *Qur’an*, the sacred book of Islam as recited by the Prophet, dreams work as a way by which God communicates with humans, as also happens in the Jewish Torah and the Christian New Testament. Dreams are also cited in some *Qur’an* passages, and in spite of appearing considerably less than in the Bible, their application rely on the ability to interpret correctly their metaphoric content, depending on the personal and circumstantial knowledge of the dreamer (Bulkeley, 2002), as highlighted by Freud (1900) and recognized by contemporary neuroscience (Ribeiro, 2019). As the Prophet said, a dream will take effect according to how it is interpreted, and a dream rests on the feathers of a bird and will not take effect unless it is related to someone. On the other hand, there are dreams that are more directly and need no interpretation, such as the famous one in which Allah tells Abraham to sacrifice his son (Bulkeley, 2002). In addition, there are strategies suggested to incubate good dreams. For example, *hadith* texts encourage practitioners to try to sleep in a state of ritual purity in order to have good dreams.

Thus, in Islam, dreams have a similar use as in biblical texts: to be interpreted, or as direct messages. In addition, in some Islamic traditions, discussions about dreams containing clear bad or “unpleasant contents” are not encouraged, because these dreams are interpreted as caused by Satan. Facing a bad dream, the dreamer is encouraged to recite the *Qŕan* and perform donations to get rid of this bad dream content, instead of discussing them with other people.

There are some references to LD in the Islamic tradition, which were made mainly by the Sufi master Ibn El-Arabi (1165–1240). El-Arabi claimed to have a strong lucid imagination and plenty of visionary experiences, such as the one in which he saw the angel Gabriel, as also happened to Muhammed. El-Arabi divided dreams into three basic types. The first are the “ordinary” dreams, which are produced by the imagination based on daily life experiences, but with symbolic content that represents our wishes, very similar to the psychoanalytic view (Jung, 1957; Freud, 1900). The second type of dream is much more important and reflects the “Universal Soul”–a kind of abstract reasoning that would reveal fundamental truths about reality, but that were also distorted by human imagination, and thus requires interpretation to unveil what the symbolic images really mean. The last type of dream involves a clear vision of divine truth with no distortions or symbolisms (Bulkeley, 2002). Regarding LD, El-Arabi believed they were also very important, and once said: “A person must control his thoughts in a dream. The training of this alertness (…) will produce great benefits for the individual. Everyone should apply himself to the attainment of this ability of such great value” (Gackenbach and LaBerge, 1988).

According to Hermansen (1997, p. 27), “The Sufi tradition specifically cultivated the preservation of some “observer faculties” during the stage of sleep by means of techniques of physical deprivation such as fasting and remaining awake throughout the night, and by exercises such as self-remembering.” A great deal of LD training has also been developed by Indian Sufi orders that migrated to the West. According to Pior Vialat Khan, the dreamer who can maintain focus and lucidity is able to work with the symbols of the World of Images, and participate with awareness in her own process of spiritual development (Khan, 1991). Another movement, the Golden Sufi center inspired by the spiritual leader Llewellyn Vaughan-Lee (1990, 1991), has incorporated dream work in their traditions. The practices involve both sharing and collective interpretation of dreams, as well as the induction of LD. There are even earlier roots of LD in Sufism, going back to medieval Islamic cultural traditions which had some contact with Hindu teachings and practices. There are certainly parallels between aspects of Hinduism and the Sufi quest to cultivate extraordinary states of consciousness, in both waking and dreaming, with the ultimate goal of a direct encounter with the deepest powers of the divine. Unfortunately, there is a lack of scientific works about these Islamic experiences.

## Spiritism

The three main Abrahamic religions are, in chronological order of foundation, Judaism, Christianity and Islam, as pointed before. However, out of these three well-known religions, there are a number of relatively minor ones, such as Spiritism, which is a Christian-based religion that was created by Allan Kardec in 1857, in France. Spiritism is found nowadays mainly in Brazil, due to the work of the “mediums” Chico Xavier, Waldo Vieira and many others. Mediums are those who can make the contact between the living ones and the spirits of the dead. Spiritism states that the human soul (or spirit) can “leave the physical body,” as in out-of-body experiences (OBE), and perform “astral projections” (Blackmore, 1982). The OBE is usually triggered by the “autoscopy” experience, whose etymology is “observing oneself.” OBE can be defined as the sight of a look-alike, that is, another self that is less real than the original self (Blackmore, 1982), or the experience of seeing one’s own body in an extra-personal space (Blanke et al., 2004).

Based on reports of autoscopy experiences during sleep, especially those in which dreamers see themselves laying on bed and sleeping, Spiritism claims that the spirit naturally detaches from the body during sleep, which would explain several aspects of dreams phenomenology. The irrational and confused aspect of dreams, for example, would be the remembrance of what the Spirit saw, but its coarse physical body would not retain the impressions grasped by the Spirit, which would explain the enormous memory gaps in dream reports. In addition, Spiritism believers claim that–during sleep–our spirit communicates with other spirits, in addition to being able to visit other worlds and have glimpses of the past and the future (de Sá and Mota-Rolim, 2015). Interestingly, some Spiritism branches affirm that LD would be the final stage before this experience of “leaving the physical body.” However, for those who believe in Spiritism, the LD would not be “real” because it is only a dream, in comparison to the astral projection. Another difference is that during LD it is possible to have control (with various degrees) over the oneiric content, which would not happen in the “true” OBE that occurs during sleep (Vieira, 2002).

Modern research has confirmed that OBE can occur during the awake state (Ehrsson, 2007), sleep (Blackmore, 1982), or dreaming (Irwin, 1988; LaBerge et al., 1988; de Sá and Mota-Rolim, 2016). According to Levitan et al. (1999), OBE can also happen during some LD, and both may share some features, such as sleep paralysis, vibrations and a sensation of floating out of body. These authors investigated the relation between OBE and LD in two studies. In the first one, the authors analyzed the content of the dream, and observed that from 107 LD episodes recorded on the lab, 10 (9.3%) were qualified as OBE. In the second study, Levitan and colleagues conducted a survey in 604 subjects, and observed that frequency of OBE was similar to that observed in the first study, which support an association between OBE and LD. The authors believe that any state that combines a high level of cortical activation with low awareness of the body has the potential to induce an OBE (Levitan et al., 1999).

OBEs are related to the function of the temporo-parietal junction, a multimodal brain region that integrates visual, tactile, proprioceptive, auditory and vestibular information (processed by occipital, parietal and temporal cortices), contributing to self-consciousness and body internal imagery (Blanke and Mohr, 2005). OBEs can be artificially induced by disrupting the temporo-parietal region with magnetic (Blanke et al., 2005) or electric (De Ridder et al., 2007) stimulation. A much simpler way to induce an OBE was developed by Ehrsson (2007), who used a glass that showed to participants the image from a camera that was positioned on their back. Standing behind the participant, Ehrsson manipulated two plastic sticks, one of which touched the participant’s chest and the other made a similar movement in front of the cameras, directing the stick to a place below them. Such synchronous movement induced a sort of cognitive dissonance, or misinterpretation: the participants felt as if their “illusory body”–created by the cameras–was their real body, thus reporting an OBE. It is very likely that OBE are involved in the practice of Spiritism.

Research like this highlights what can be learned by applying scientific methods to the study of dreaming in religious contexts. This is especially true when we look beyond the major world religions of Hinduism, Buddhism, Judaism, Christianity, and Islam. For smaller religious movements like Spiritism, dreams are a very appealing resource. Dreaming can provide a direct, deeply personalized means of accessing powerful spiritual energies and modes of higher awareness. The anthropology of dreaming offers evidence of LD in small-scale societies and indigenous communities around the world (Lohmann, 2003). In these traditions the emphasis of LD practice is often the shamanic work of healing, prophecy, and spiritual empowerment. During shamanic rituals, it is often observed the use of substances that alter the consciousness, such as Ayahuasca, and there is a close phenomenological relationship between dreams, specially LD, and the psychedelic experience (Kraehenmann, 2017). This active, purposeful approach to dreaming is closer to Hinduism and Buddhism than to the Abrahamic faiths, but with less interest in metaphysics and more in the pragmatic challenges of this world.

## Conclusion and Perspectives

In the three Abrahamic monotheisms–i.e., Judaism, Christianity, and Islam–the focus is mainly on the interpretation of dreams to understand the present and predict the future. On the other hand, in traditional Indian religions such as Buddhism and Hinduism, there are specific and well-documented techniques to induce LD. This suggests that while the monotheistic religions are related to understanding the will of God, in the polytheistic or atheistic beliefs from India there is a focus on the cultivation of self-awareness. In indigenous cultures and smaller religious traditions like Spiritism, the approach to LD tends to be less abstract and more focused on responding to personal and communal challenges. In light of these differences, it would be interesting to investigate whether people’s religious beliefs and practices correlate with their frequency of LD. Based on the foregoing historical review, we could hypothesize that members of Abrahamic traditions have fewer LD experiences, and members of traditions like Hinduism, Buddhism, and Spiritism have more LD. However, to pursue this line of investigation would also require accounting for the considerable number of people who do not affiliate with any religious tradition. The non-religious constitute a sizable portion of the population in modern societies, and it is possible that LD frequencies are higher among non-religious people than among those who are part of a formal religious tradition.

This historical review makes clear that LD is not a modern invention. Human awareness of, and experimentation with, LD goes back thousands of years. This evidence supports the idea that LD is a natural, though somewhat rare, feature of the human sleep cycle. This review also highlights the cross-cultural fact that LD regularly elicits spiritual responses, even among people who are not formally religious. There is something about the appearance of consciousness in dreaming that almost automatically stimulates feelings of deep wonder about the fundamental nature of mind and cosmos. People today continue to express similar feelings about their experiences of LD. One of the challenges for contemporary scientific researchers is how to explain the powerful impact of LD at the highest levels of conceptual thought, from the beginnings of history right into the present day.

## Author Contributions

All authors listed have made a substantial, direct and intellectual contribution to the work, and approved it for publication.

## Conflict of Interest

The authors declare that the research was conducted in the absence of any commercial or financial relationships that could be construed as a potential conflict of interest.
